# Beyond the Knife in Renal Cell Carcinoma: A Systematic Review—To Ablate or Not to Ablate?

**DOI:** 10.3390/cancers15133455

**Published:** 2023-06-30

**Authors:** Harshani Green, Alexandra Taylor, Vincent Khoo

**Affiliations:** 1 Royal Marsden Hospitals NHS Foundation Trust, London SW3 6JJ, UKvincent.khoo@rmh.nhs.uk (V.K.); 2 Institute of Cancer Research, London SW7 3RP, UK; 3 Department of Medical Imaging and Radiation Science, Monash University, Clayton, VIC 3800, Australia; 4 Department of Medicine, University of Melbourne, Parkville, VIC 3010, Australia

**Keywords:** renal cell carcinoma, RCC, ablation, ablative therapies, stereotactic radiotherapy, SABR, SBRT, radiofrequency ablation, cryotherapy

## Abstract

**Simple Summary:**

Over the last 10 years, there has been a vast increase in the use of targeted and immunotherapy drugs in the management of advanced kidney cancer. As patients are living longer with cancer that has spread, the natural history has altered, with problems of poor tumour control in one or a few areas only. Local surgical removal of the tumour(s) frequently cannot be applied due to technical challenges or patient factors. Thermal ablation or stereotactic body radiotherapy (SBRT) may be utilised. Thermal ablation is a minimally invasive treatment where heat is generated and passed via a probe into the tumour to cause cancer cell death. SBRT is a non-invasive treatment where high radiobiological doses of radiation are precisely delivered in one, or a few, treatments to kill cancer cells. This systematic review aims to evaluate the existing evidence for these treatments to help improve personalised care for individual patients.

**Abstract:**

Intensified systemic therapy in metastatic renal cell carcinoma (mRCC) has led to improved patient outcomes. Patients commonly require local control of one or a few metastases. The aim was to evaluate metastasis-directed ablative therapies in extracranial mRCC. Two databases and one registry were searched, using the Preferred Reporting Items for Systematic Reviews and Meta-Analyses (PRISMA) approach, for all prospective and matched-pair case–control mRCC studies of radiofrequency ablation (RFA), cryotherapy, microwave ablation (MWA), and stereotactic body radiotherapy (SBRT). Eighteen studies were identified. Fourteen investigated SBRT in 424 patients. Four thermal ablation studies were identified: two cryotherapy (56 patients) and two RFA studies (90 patients). The median participant number was 30 (range 12–69). The combined median follow-up was 17.3 months (range 8–52). Four SBRT studies reported local control (LC) at 12 months, median 84.4% (range 82.5–93). Seven studies (six SBRT and one cryotherapy) reported an LC rate of median 87% (79–100%). Median overall survival (OS) was reported in eight studies (five SBRT, two cryotherapy, and one RFA) with a median of 22.7 months (range 6.7–not reached). Median progression-free survival was reported in seven studies (five SBRT, one cryotherapy, and one RFA); the median was 9.3 months (range 3.0–22.7 months). Grade ≥ 3 toxicity ranged from 1.7% to 10%. SBRT has excellent local control outcomes and acceptable toxicity. Only four eligible thermal ablative studies were identified and could not be compared with SBRT. Translationally rich definitive studies are warranted.

## 1. Introduction

### 1.1. Description of the Condition

#### 1.1.1. Renal Cell Carcinoma (RCC)

RCC is the seventh most common cancer in the UK and accounts for 4% of all new cancer cases [1]. Its incidence is projected to increase by 26% by 2035. Metastatic disease is present in 30% of RCC patients at diagnosis, amounting to approximately 3960 cases per year. For patients with localised disease treated with curative intent, nearly 30% will subsequently relapse with metastatic disease. Renal cancer has an unpredictable heterogenous biology and can present with a relatively indolent process in some, and with an aggressive pattern in others.

#### 1.1.2. Systemic Therapy in the First-Line Treatment Setting

The outcomes from multiple first-line randomised controlled trials (RCTs) (NCT00083889, JAVELIN 101, KEYNOTE-426, CheckMate 214, and CheckMate 9ER), all of which investigated treatment-naïve patients with a clear cell component, have dramatically changed the treatment landscape in RCC with substantial improvements in survival. Treatment largely consists of tyrosine kinase inhibitors (TKI), immunotherapy (IO), mammalian target of rapamycin inhibitors (mTOR-I), and their combinations. Median progression-free survival (PFS) has significantly improved from around 5 months with interferon therapy to 8.4 months (range 7.0–11.1) with systemic targeted agents and subsequently to 14.7 months (range 11.6–23.9 months) using combination regimes of targeted and IO agents [2,3,4,5,6,7]. Median overall survival (OS) for a first-generation TKI, sunitinib, ranges across these studies from 26 months to not reached [2,3,4,5,6,7]. The median OS for other treatments were generally not reached within their respective median follow-ups, which ranged from 9.9–30.6 months [3,4,5,6,7]. However, there is still room for further improvement of PFS and OS.

#### 1.1.3. Systemic Therapy in the Second-Line Treatment Setting or More

In second-line treatment, three RCTs (AXIS, METEOR, and Checkmate 025) reported median OS ranges from 17.1 to 25.9 months with corresponding median PFS ranging between 3.8 and 8.3 months [8,9,10]. Checkmate 025 allowed 1–2 prior lines of treatment. These results are similar to the survival outcomes seen within this review. There are limited further prospective, randomised data in the third-line or more setting. The TIVO-3 phase 3 RCT investigated 350 patients with advanced clear cell RCC in 350 patients who had progressed on two or three prior regimens with a median PFS of 5.6 months with tivozanib vs. 3.9 months in the sorafenib arm [11]. At this advanced stage in a patient’s treatment pathway, more modest benefits in PFS are arguably much more clinically significant to patients than in the first-line setting.

#### 1.1.4. Metastasectomy

Metastasectomy involves the surgical resection of metastatic sites of disease. Systematic reviews and meta-analyses of retrospective data report a positive benefit of metastasectomy for survival, including PFS and OS, as well as offering potential delays in systemic therapy and reduced exposure to adverse events [12,13,14,15,16,17]. OS ranges between 29.6 and 142 months for metastasectomy compared to no metastasectomy at 8–27 months. However, it should be noted that surgery is generally used in optimally selected patients who are fit, have limited comorbidities, and have a limited number of metastases. It is thought that a combination of targeted agents in conjunction with metastasectomy may result in better survival outcomes than metastasectomy alone for mRCC [13,14,17].

#### 1.1.5. Active Surveillance (AS)

There appears to be a subset of patients with mRCC who demonstrate a relatively indolent pattern of metastatic growth; hence, there is an argument to implement AS, i.e., holding initiation of active treatment with regular imaging and clinical surveillance. Two prospective studies investigated initial AS in mRCC with the potential benefit of delaying time to systemic therapies and, as such, delaying associated toxicity [18,19]. However, in both of these studies, prior surgery (nephrectomy or metastasectomy) and prior radiotherapy were permitted. In one non-randomised phase 2 study of 48 asymptomatic treatment-naïve patients, the median time for surveillance until initial systemic therapy was 14.9 months after a median follow-up of 38.1 months [18]: 23% favourable risk (FR), 75% intermediate risk (IR), and 2% poor risk (PR). Two patients developed new central nervous system (CNS) diseases during the surveillance period, although no other patients had symptomatic progression during this period. Multivariate analysis showed that a higher number of metastatic sites and a higher International Metastatic RCC Database Consortium (IMDC) risk score were associated with a shorter surveillance period. This highlights that careful selection criteria for AS is warranted and CNS imaging should form part of routine surveillance.

Interestingly, in a real-world observational study of 143 patients undergoing AS, 43% of the AS cohort included patients who developed metastases within 1 year of initial diagnosis, which has generally been considered an indicator of a very poor prognosis [19]. On subgroup analysis, OS in these patients was similar to that of patients who developed metastases much later; however, patients chosen for AS generally had low-volume and asymptomatic disease. Inevitably, these studies had considerable selection bias with very low proportions of high-risk cohorts.

### 1.2. Description of Interventions/How These Interventions May Work

TRACERx RENAL explored the evolutionary patterns of metastases in RCC. The study demonstrated that metastatic sites were more homogeneous and harboured fewer driver somatic alterations than their matched primary tumours [20]. The majority of driver mutations were accumulated in the primary rather than de novo within the metastasis. Local treatment of poorly-responding or residual sites alongside systemic therapy is likely to be of benefit.

Metastasis-directed local ablative therapies are targeted treatments which aim to destroy a tumour and/or its function without surgical resection. Such interventions are described in this section.

#### 1.2.1. Thermal Ablative Therapies

These are minimally invasive procedures where the approach can be percutaneous, endoscopic, or surgical. Local anaesthesia can be used, but general anaesthetic is often indicated. The different forms of thermal ablation are listed below.

##### Radiofrequency Ablation (RFA)

Percutaneous image-guided RFA is thought to cause damage to cancer cells by passing radiofrequency energy, consisting of an alternating electrical current in the frequency of radio waves, through an electrode. This then produces heat, which coagulates and destroys the tumour tissue in the target area [21]. The energy from the electrode tip produces a temperature that is proportional to the square of the radiofrequency current, which in turn decreases as the square of the diameter from the electrode [22]. RFA has shown excellent results for small <3 cm tumours in the liver, lung, or kidney. However, higher local recurrence has been shown with the percutaneous approach for larger tumours as it requires overlapping spheres, which may lead to incomplete necrosis. It is also likely not as beneficial in tumours in close proximity to major vessels, such as the portal vein. The ‘heat-sink’ effect is caused by cooling due to flowing blood in nearby blood vessels [23].

##### Cryotherapy

Cryotherapy involves ‘freezing’ of the tumour via an image-guided probe which delivers liquid nitrogen or argon gas [24,25,26]. This is associated with the formation of ice crystals, which directly damage cancer cells. Freezing is thought to cause damage to the tumour’s microvascular system via endothelial damage, resulting in oedema, inflammation, and thrombosis.

##### Microwave Ablation (MWA)

MWA involves a probe, attached to a microwave generator, which delivers microwave energy, thereby producing heat to induce tissue necrosis and tumour cell death. Multiple pulses of energy can be delivered in one session; however, for larger tumours, multiple needle electrodes are required. Potential advantages of MWA over RFA include the ability to achieve higher temperatures (>100 °C) less affected by the ‘heat-sink’ effect of nearby blood vessels [27].

#### 1.2.2. Stereotactic Body Radiotherapy (SBRT)

Radiotherapy is a non-invasive local treatment using X-rays to kill cancer cells. SBRT is a highly targeted and precise type of radiotherapy which delivers a high radiobiological dose in one or a few treatments with the aim of destroying a tumour. RCC has traditionally been considered to be radioresistant, but this concept is now outdated. There is a large spectrum of treatment responses to radiotherapy due to RCC’s inherent disease heterogeneity. It is acknowledged that the use of low-dose radiotherapy for symptom palliation can be effective. Since the introduction of modern advances in both precision radiotherapy and image-guidance techniques, there is now good evidence to support the view that SBRT can overcome this relative radioresistance with improved local control (LC) [28,29]. There are several proposed mechanisms behind this enhanced effect including affecting the microvascular infrastructure through the ceramide pathway to induce endothelial apoptosis in contrast to oxygen-dependent deoxyribonucleic acid (DNA) damage with conventional radiotherapy [30]. As RCC is a very vascular tumour, this is likely to be a more effective pathway for tumour kill. From xenograft and in vitro models, the alpha beta ratio has been described as 2.6 to 6.92 [31]; as such, there is likely increased tumour kill with higher dose per fraction as used in SBRT [32].

### 1.3. Why It Is Important to Do This Review

In the metastatic setting, the use of systemic targeted and IO agents has altered both management and natural history with significant improvements in disease-free survival (DFS) and OS. However, these treatments are associated with high levels of treatment-related toxicity; grade ≥ 3 adverse events have been reported at rates of 55% to 83.1% [2,3,4,5,6,7]. Side-effects of targeted agents may include, but are not limited to, diarrhoea, skin effects, and lethargy, whereas more inflammatory side-effects are seen with IO including pneumonitis, colitis, and hormone dysfunction. Furthermore, as patients are living longer, the patterns of failure have also altered with more RCC patients presenting with issues of LC from poorly responding and progressive local disease or metastasis [33,34,35]. Many patients cannot have surgical resection, due to either technical feasibility or patient factors. Overall, the survival of patients with mRCC remains poor with reported 5-year OS rates of 10–14%; thus, evaluation of other treatment strategies is warranted to improve patient outcomes [1].

**Figure 1:**
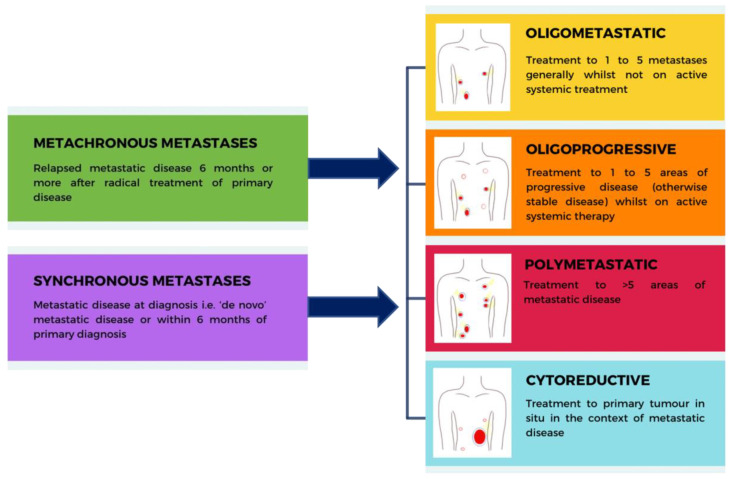
Definitions of different settings of metastatic cancer. The pictures show a generalised representation of areas of renal cell carcinoma within the body (red areas) in each setting, which may be treated with ablative techniques. Sites of metastatic disease are not limited to these areas and will vary with individual patients.

It is recognised that not all metastatic diseases should be considered alike. Low-volume and limited metastatic diseases, i.e., oligometastases, may have a different natural history and prognosis from high-volume widespread metastatic diseases, i.e., polymetastases [36]. Figure 1 outlines the definitions of different metastatic settings.

SBRT is increasingly used to control low-volume metastases, as well as progressive and persistent sites of mRCC. In a recent basket meta-analysis to evaluate SBRT in OM disease, 21 prospective studies comprising 943 patients and 1290 OMs were included [37]. It reported clinically acceptable rates of grade 3–5 toxicity with an excellent LC of 94.7% (95% CI, 88.6–98.6%) [37], and the 1-year OS was 85.4% (95% confidence interval (CI), 77.1–92.0%). Median follow-up was 16.9 months [37]. Eight-year outcomes were recently reported from a prospective randomised basket phase 2 trial, SABR-COMET, of 99 patients with metachronous OM disease [38,39]. An OS benefit was seen with SBRT versus palliative radiotherapy; the 8-year OS was 27.2% and 13.6%, respectively (HR, 0.50; 95% CI 0.30–0.84; *p* = 0.008). However, this study was designed as a phase 2 trial and was not a definitive phase 3 RCT. The trial reported three treatment-related deaths (median follow-up 26 months for SBRT). After an extended median follow-up of 5.7 years, there were no new major toxicity signals, and grade ≥ 2 toxicity was 30.3% for SBRT and 9.1% in the control group.

Percutaneous ablation of primary RCC is now a well-recognised treatment option, particularly in the treatment of T1a renal masses. It has a relatively low cost, short duration, and low complication rate in comparison to surgical techniques [27]. There is increasing data to support the safety of thermal ablation in the lung, kidney, and liver [26,40,41]. Although these techniques are increasingly being utilised in the metastatic setting, there are much less data to evaluate its efficacy.

#### One Treatment Strategy May Not Fit All

In studies investigating ablative therapies that permit any histological subtypes, proportions of mRCC patients are often not reported or represent less than 1–5% of the recruited population [38,39]. A phase 2 single institution trial (PROMISE-004: CURB) recruited 107 metastatic non-small-cell lung cancer (mNSCLC) and metastatic breast cancer (mBC) patients with 1–5 OP lesions randomised to SBRT with palliative standard of care (SOC) versus palliative SOC alone [42]. Interim analysis reported a PFS benefit of 22 weeks for the combination arm compared to 10 weeks for the SOC arm (*p* = 0.005). The authors outlined that this effect was largely driven by mNSCLC patients where median PFS for the combination therapy was 44 weeks versus 9 weeks for the SOC arm (*p* = 0.004). There was no difference in median PFS in the mBC group. This is consistent with results reported by a multicentre randomised phase 2/3 trial in oligometastatic (OM) breast cancer with controlled locoregional disease and ≤4 OM (NRG-BR002) [43]. This study of 125 mBC patients did not show improvement in PFS or OS for the addition of ablative therapy using either SBRT or surgery. This suggests that the benefits of ablative therapy can differ with histology; therefore, evaluation in mRCC is warranted.

### 1.4. Objectives

- To review the current role of metastasis-directed ablative therapies in the management of adult extracranial metastatic renal cell carcinoma inclusive of efficacy outcome measures and toxicity of different treatment modalities.
- To evaluate different ablative techniques including thermal ablation (including RFA, cryotherapy, and MWA) and SBRT.

## 2. Materials and Methods

This was a PROSPERO-registered systematic review. The full protocol can be accessed at https://www.crd.york.ac.uk/prospero/ [registration number CRD42023395322]. Last accessed on 26 June 2023.

### 2.1. Criteria for Considering Studies for This Review

#### 2.1.1. Types of Studies

RCT or quasi-randomised trials, prospective nonrandomised (single-arm) studies, and matched-pair case–control studies with at least 10 patients were considered.

#### 2.1.2. Population

Adult patients (18 or over) with metastatic extracranial RCC were considered.

#### 2.1.3. Comparisons

No comparisons were applicable as this was a descriptive systematic review.

#### 2.1.4. Outcome Measures

Toxicity was measured using Grade 3–5 adverse effects. LC was measured using the LC rate at 1 year, 2 years, and 5 years (if available). Response was measured using the overall response rate (ORR). Survival was measured using the OS rate at 1 year or median OS and PFS at 1 year or median PFS. The median time to next systemic therapy (TTNT) and validated patient-reported outcome measures (PROMs) were also measured.

### 2.2. Search Methods for Identification of Studies

#### 2.2.1. Search Databases/Registries

- MEDLINE
- The Cochrane Library
- Clinical Trials.gov

#### 2.2.2. Search Strategy

The search strategy involved the terms outlined below. Terms listed were further expanded using tools such as ‘MESH’ for a comprehensive search on 18 January 2023. Studies were limited only to English Language; there was no time limit applied.

Keywords and outline of search strategy:

[Renal cell carcinoma ‘OR’ RCC]‘AND’
[Metastasis ‘OR’ Metastases ‘OR’ Metastatic]
‘AND’
[Ablation ‘OR’ Ablate ‘OR’ Ablative ‘OR’ Thermal ablation ‘OR’ Catheter ablation ‘OR’ Radiotherapy ‘OR’ Irradiation ‘OR’ Radiosurgery ‘OR’ Stereotactic body radiotherapy ‘OR’ SBRT ‘OR’ SRT ‘OR’ Stereotactic radiotherapy ‘OR’ Stereotactic ablative radiotherapy ‘OR’ SABR ‘OR’ Radiofrequency ablation ‘OR’ RFA ‘OR’ Cryotherapy ‘OR’ Cryoablation ‘OR’ Microwave]

#### 2.2.3. Inclusion Criteria

- >18 years
- English language

#### 2.2.4. Exclusion Criteria

- Retrospective (unless matched-pair case–control)
- Terminated/withdrawn studies
- Non-RCC histology
- Basket studies (non-histology specific) with fewer than 10 RCC participants or that did not report how many RCC participants
- Review articles, editorials, and case reports
- Paediatric cohorts
- Intracranial studies
- Radiology studies

### 2.3. Data Collection and Analysis

#### 2.3.1. Selection of Studies

Titles and abstracts of clinical studies were imported into the reference manager software (EndNote 20.4). Results from https://www.clinicaltrials.gov database were manually screened [Accessed 18 January 2023]. This list was screened by one author, and the full text was retrieved as required.

#### 2.3.2. Data Extraction and Management

Data were extracted from each identified paper. The extracted data included information on trial design participants, type of intervention, and outcome measures. A descriptive review was carried out.

A list of titles and abstracts of clinical studies were imported into the reference manager software (EndNote). The title list was screened by one author (H.G.). Abstracts were then reviewed for all relevant studies, and the full text was retrieved as required for all potentially relevant studies. Full papers and relevant abstracts were formally assessed by two authors (H.G. and V.K.) to ensure they met the inclusion criteria for the review. Studies that were considered borderline for inclusion in the review were discussed among three authors (H.G., V.K., and A.T.) as part of a peer-review process. Where more than one report contained the same datasets, the largest dataset was used.

Data were extracted from each identified eligible paper. The extracted data included information on publication details (citation, authors, title, and year), trial design participants (age, sex, tumour pathology, and metastatic setting), type of intervention, and outcome measures (as above). A descriptive review was carried out (H.G.) with peer review (V.K. and A.T.). Data were recorded in an Excel spreadsheet.

#### 2.3.3. Strategy for Data Synthesis

At least five eligible studies were required to be identified for data synthesis. Findings were reported in keeping with the Preferred Reporting Items for Systematic Reviews and Meta-Analyses (PRISMA) guidelines [40]. There was a paucity of randomised data to carry out a formal quantitative assessment/meta-analysis of the beneficial effects of local ablative therapy. Therefore, studies were described using a narrative approach to review LC, survival outcomes (where available), and harms of treatment. Study information, LC rates, and toxicity for all eligible studies were summarised in a table format, listed according to treatment technique, thermal ablative therapies, and SBRT. Medians and ranges were included where appropriate. Studies were described according to relevant metastatic settings, e.g., OM, oligoprogression (OP), polymetastatic (PM), and cytoreductive (CR). Ongoing studies were identified and listed in table format. A risk-of-bias assessment was carried out by H.G. and V.K. for all eligible full papers (ROBINS-I tool for non-randomised studies).

## 3. Results

### 3.1. Description of Studies

#### 3.1.1. Included Studies

**Figure 2:**
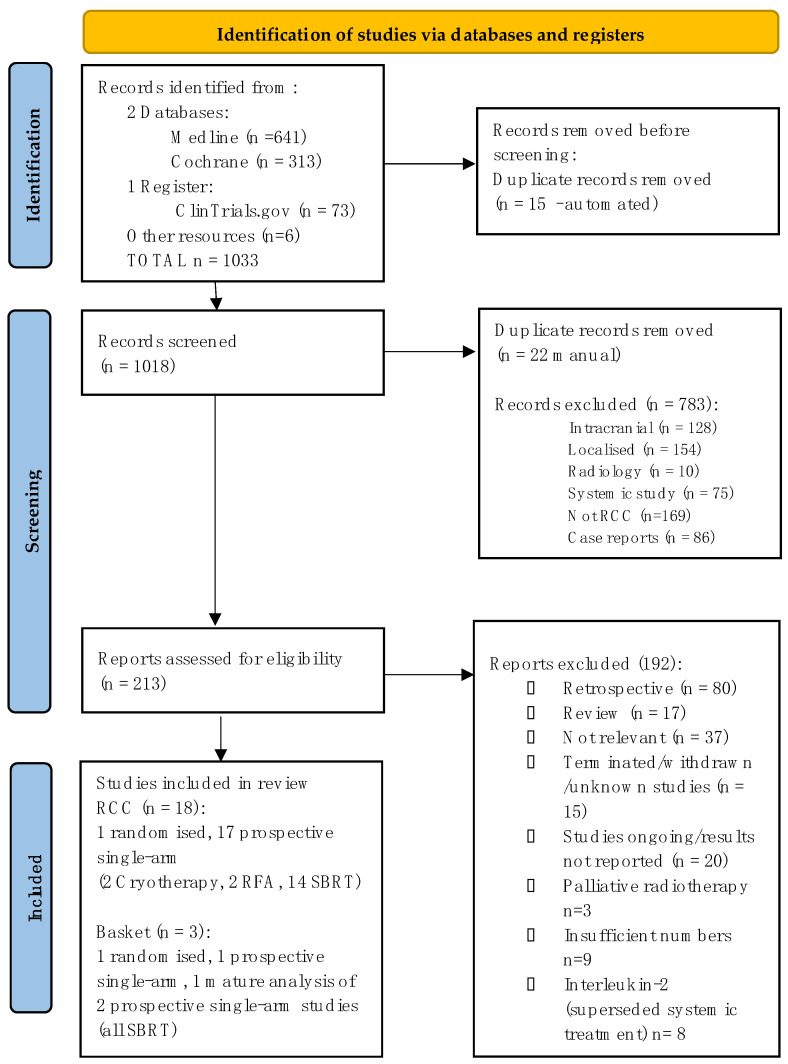
PRISMA flow chart for identification of eligible studies [44].

A summary of studies identified through a literature search from 18 January 2023 is displayed in a PRISMA flow chart (see Figure 2 and Supplementary Materials). A total of 1033 records were identified, of which 1018 were screened; 213 reports were identified as appropriate for eligibility assessment. Eighteen prospective studies evaluating metastasis-directed therapies in RCC were identified: one randomised and 17 single-arm.

##### RCC SBRT Studies (See Table 1a) and RCC Thermal Ablation Studies (See Table 1b)

Table 1a summarises all eligible RCC SBRT studies (*n* = 14), and Table 1b shows all RCC thermal ablative studies (*n* = 4; two cryotherapy and two RFA). No eligible MWA studies were identified. The total number of patients treated was 570; 56 were treated with cryotherapy, 90 were treated with RFA, and 424 were treated with SBRT.

In OM, one cryotherapy study and two SBRT studies were identified. In the two SBRT studies, 100% of patients had nephrectomy. In the OP setting, two SBRT studies were identified, which contained both synchronous and metachronous metastatic patients. In the PM setting, one cryotherapy, one RFA, and eight SBRT studies were identified. In the CR setting, one RFA study, one SBRT study, and one SBRT + surgery study were identified.

##### Basket Studies of Multiple Histologies (See Table 1c)

Six basket spinal SBRT studies were identified that reported over 10 RCC patients each; one was randomised (see Table 1c). Two of these papers were mature analyses of previous studies. As per protocol, the largest cohorts were eligible for inclusion (*n* = 3), and overlapping smaller cohort reports were not included in the summary (*n* = 3). One study evaluated combination IO–SBRT, and two were prospective spinal SBRT studies.

##### Ongoing Trials (See Table 2)

Table 2 provides a summary of ongoing trials of metastasis-directed therapies in mRCC. A total of 20 prospective studies were identified: 11 mRCC radiotherapy trials, 7 basket radiotherapy studies (including three RCTs), and 2 basket thermal ablation trials. No mRCC thermal ablative studies were identified. Around half of all ongoing studies are estimated to be completed by 2025.

#### 3.1.2. Excluded Studies

Studies investigating metastasis-directed treatment of intracranial RCC were not included, as the biology of this disease setting, as well as its management, is very different from extracranial metastatic disease (*n* = 128). Eight studies in combination with interleukin-2 (IL2) were excluded as this treatment has been superseded. Retrospective studies were excluded unless they were matched-pair case–control studies, as the authors agreed that they would have significant risk of both selection and recall bias (*n* = 78). Studies which had fewer than 10 patients or basket studies which included either <10 RCC patients or did not report the numbers of RCC patients were not included (*n* = 9). The authors agreed these were likely to represent very early experience with the techniques, e.g., technical reports, and would have insufficient numbers to be able to review clinical outcomes.

#### 3.1.3. Risk of Bias in Included Studies

Of the two randomised studies identified, only one could be formally assessed for bias, as the other has been published in only abstract format to date [45]. In a randomised feasibility study of tremelimumab with or without cryotherapy in RCC, participants were randomised; however, the randomisation method was not reported [46]. The two treatment arms had comparable numbers. The study was not blinded, which is acceptable due to the nature of the interventions. The paper reported demographics, numbers, and stoppages due to adverse events. However, it did not adequately report the anatomical sites of metastatic disease treated with cryotherapy and was assessed as having a moderate reporting bias. The authors identified that it was not formally powered for efficacy outcomes with a small cohort of 30 cases.

Of the remaining non-randomised eligible studies, these were assessed using the ROBINS-I tool as per the Cochrane guidelines. One study was not assessed as it was only available in abstract form [47]. Results are summarised in Table 3.

### 3.2. Effects of Interventions

#### 3.2.1. Overall Numbers

Of the 18 studies in RCC, patient numbers were generally small; the median number of participants was 30 (range 12–69). A further 82 RCC patients were identified in three basket studies of 205 patients in total (40%).

Note that all descriptive statistics below are for RCC studies alone; basket studies (Table 1c) were not included. See Table 1a,b for full data summary.

#### 3.2.2. Demographics/Patient Characteristics

The median age was 62 (range 54.5–66.8). Of the 15 studies that reported the sex of participants, a median of 79% were male (range 67–88.9%). Four studies reported ethnicity, with a median of 71% patients being Caucasian (range 0–90).

The median percentage of clear cell patients was 93.5% from 10 studies (range 62–100). In the seven studies which reported the proportion of risk groups by the IMDC or Memorial Sloan–Kettering Cancer Centre (MSKCC), the median proportion of FR/good-risk patients included in the studies was 25% (range 8–56%), in contrast to 68% for IR (range 44–80%) and 7.5% for PR/high risk (range 0–25%).

#### 3.2.3. Follow-Up

Thirteen studies (11 SBRT and two thermal ablation) reported median follow-up time; the median was 17.3 months (range 8–52). Follow-up for the two thermal ablation studies alone was 16 months and 29 months (Table 1b).

#### 3.2.4. Local Control

Four SBRT studies reported LC at 12 months, median 84.4% (range 82.5–93). Seven studies (six SBRT and one cryotherapy) reported the LC rate, with a median of 87% (79–100%), as well as a follow-up median of 16.8 months (range 10.4–37).

#### 3.2.5. Overall Survival

Median OS was reported in seven studies (four SBRT, one RFA, and two cryotherapy) with a median of 22.7 months (range 6.7–32.3 months); median follow-up of these seven studies was 22 months (range 13.1–29 months). Median OS was not reached in one further SBRT study after a median follow-up of 10.4 months.

The range of median OS for the five radiotherapy studies that reported this was 6.7 months–not reached (Table 1a). Median OS for the three thermal ablative studies which reported this outcome ranged from 22.7 to 32.3 months (Table 1b).

#### 3.2.6. Progression-Free Survival

Median PFS was reported in seven studies (five SBRT, one RFA, and one cryotherapy); the median was 9.3 months (range 3.0–22.7 months); the median follow-up of six of these studies was 14.7 months (range 10.4–29 months).

Median PFS for SBRT studies alone ranged from 5.6 to 22.7 months (Table 1a). Median PFS in two thermal ablative studies was 3.0 and 13.4 months (Table 1b).

#### 3.2.7. Severe Toxicity

The incidence of grade ≥ 3 toxicity ranged from 1.7% to 10% for local metastasis-directed ablative therapies (see Table 1a,b). One study reported a possible death that may or may not have been related to SBRT treatment in a case of a large pleural lesion (see Table 1a). No other ablation-related deaths were reported. In systemic combination studies, reported grade ≥ 3 toxicity ranged from 0% to 55% (Table 1a,b).

**Table 1:** (**a**) SBRT studies in metastatic extracranial RCC [47,48,49,50,51,52,53,54,55,56,57,58,59,60]. (**b**) Thermal ablation studies in metastatic extracranial RCC—cryotherapy and radiofrequency ablation [46,61,62,63]. (**c**) SBRT basket studies which included metastatic extracranial RCC patients (No thermal ablative basket studies identified) [64,65,66,67,68].

Trial Author	Setting	Study Design	Patient Numbers	Risk Group	Nephrectomy	Pathology (% Clear Cell)	Systemic Treatment	Treatment Regime	FU Median (Months)	Local Disease Control	OS	PFS	Toxicity
(**a**)													
Tang et al., 2021 [48]	OM	Phase 2 single-arm	30	IMDC: favourable 47%, intermediate 50%, poor 3%	100%	100%	Up to 1 prior line; no concurrent systemic treatment permitted	Sequential RT to all OM (SBRT ≤5 fractions with ≥7 Gy/f, If IMRT 52.5–70 Gy in 10–15 f); 43.3% 2nd course of radiotherapy	17.5	LC 97%. 1-year systemic therapy-free survival 82% (95% CI 70–98)	NR	Median PFS 22.7 m	≥Grade 3 10% (back pain/muscle ache, hyperglycaemia)
Siva et al., 2022 (RAPPORT) [49]	OM	Single-arm	30 (83 lesions)	IMDC: favourable 56%, intermediate 44%, poor 0%	100%	100%	Pembrolizumab	SBRT to all OM + IO 77%—20 Gy in 1f, 23%—30 Gy in 10f	28.0	DCR 83%	12 m OS 90%, 24 m OS 74%	12 m PFS 60%, 24 m PFS 45%	13% overall Grade 3 toxicity, 6.5% Grade 3 toxicity attributable to IO + SBRT
Hannan et al., 2021 [50]	OP	Phase 2 single-arm	23 (37 lesions)	IMDC: favourable 25%, intermediate 75%, poor 0%	60% CN, 35% RN (60% synchronous mets)	90%	Up to 4 lines TKI, IO, mTOR-I	Sequential SBRT to all OP lesions (8.1% kidney). 25 Gy × 1f, 12 Gy × 3 f, 8 Gy × 5f	10.4	LC 100%, extended duration of the ongoing systemic therapy by >6 m in 70%, 95% CI: 49.9–90.1)	Median OS not reached	Median PFS 11.1 m 12 m 79%	Grade 3 5%
Cheung et al., 2021 [51]	OP	Phase 2 single-arm	37 (57 lesions)	IMDC: favourable 32%, intermediate 68%, poor 0%	100%	65%, 35% clear cell component	TKI and IO	SBRT to 1–5 OP lesions. Median 72 Gy BED	11.8	12 m LC 93% TTNT 12.6 m	12 m OS 92%	Median PFS 9.3 m 12 m 84.7% 24 m 50.8%	No grade ≥ 3 toxicity
de Wolf et al., 2017 [52]	PM	Phase 1 dose-escalation	13	MSKCC good 31%, intermediate 54%, high 0%	100%	NR	TKI	SBRT to largest lesion + TKI (24, 30, 36 Gy in 3f)	10.9	12 m LC 83%	NR	Median PFS 6.7 m	No DLTs seen at 24 and 30 Gy; at 36 Gy—single DLT Gd 4 hypoglycaemia; Gd 3–4 pazopanib toxicity 38%
Masini et al., 2022 (NIVES) [53]	PM	Phase 2 single-arm	69	IMDC: favourable 26%, intermediate 65%, poor 9%	77%	80%	Nivolumab	SBRT to largest site, 30 Gy in 3f	26.0	DCR 85% ORR 17%	Median OS 20 m	Median PFS 5.6 m	All grade 3 toxicity outside of irradiated area
Dengina et al., 2019 (VOLGA) [54]	PM	Phase 1b single-arm	17 (17 lesions)	NR	24% CN, 71% RN		TKI and IO	If stable for 4 months on systemic treatment, SBRT to 1 of 2 lesions in the same organ/compared to control lesion; 65% BED >100 Gy	8.0	ORR 76%	NR	NR	No grade ≥ 3 toxicity
Hammers et al., 2020 (RADVAX abstract) [47]	PM	Single-arm	25	IMDC: favourable 8%, intermediate 80%, poor 12%	77%	100%	Ipilimumab/nivolumab	SBRT to 1–2 lesions + IO (50 Gy in 5f)	NR	ORR 56%	NR	NR	40% requiring steroids for dual IO toxicity; no grade 3 toxicity attributed to SBRT
Nguyen et al., 2010 [55]	PM	Single-arm	48 (55 lesions)	NR	100%	NR	NR	SBRT to spine; 24 Gy/1f, 27 Gy/3f, 30 Gy/5f	13.1	12 m spinal PFS 82%; 52% pain-free at 12 m (23% at baseline)	Median OS 22 m 12 m OS 72%	NR	4% grade ≥ 3 toxicity (pain, anaemia)
Sohn et al., 2014 [56]	PM	Matched-pair retrospective	26	NR	NR	NR	NR	61.7 Gy (± 19.6) SBRT and 32 ± 8.4 for RT arm	NR	12 m LC 85.7% SRS 12 m 29% with RT VAS change SBRT 4.4 ± 2.3 VAS change RT 2.8 ± 2.5	NR	PFS benefit reported for SBRT vs. RT	No grade ≥ 3 toxicity 15.3% fracture SRS, 0% RT
Gerszten et al., 2005 [57]	PM	Single-arm	48 (60 lesions)	NR	NR	NR	NR	SBRT to spine, mean 20 Gy, 70% retreatments	37 m	87.5% local control (reported for 8 lesions), pain control 89%	NR	NR	No significant toxicity reported in immediate post-procedural timeframe
Svedman et al., 2006 [58]	PM	Phase 2 single-arm	30 (82 lesions)	NR	83%	NR	No treatment 4 weeks before SBRT	Sequential SBRT allowed (70.1% lung, 11.2% kidney) (8 Gy × 4, 10 Gy × 4, 15 Gy × 2, 15 Gy × 3)	52 m alive patients, 22 m dead patients	79%	Median OS 32 m	NR	1 possible Gd 5 toxicity (large pleural lesion)
Correa et al., 2018 [59]	CR	Phase 1 dose-escalation	12	IMDC: favourable 8.3%, intermediate 66.7%, poor 25%	Not applicable	75%	50% no ST; adjuvant TKI/ mTOR-I permitted	SBRT to whole kidney (25 (*n* = 3), 30 (*n* = 6), 35 Gy (*n* = 3) in 5 f)	22.0	17.3% median size reduction	Median OS 6.7 m	NR	Single grade 3 DLT at 30 Gy (group repeated); 2 grade 3 DLTs in 35 Gy cohort (MTD = 35 Gy); G3 events (2 fatigue, 1 bone pain); no significant reduction in GFR at 12 weeks
Singh et al., 2022 [60]	CR	Single-arm	16		*n* = 3 partial, *n* = 11 total post SBRT	75%	None	SBRT to kidney lesion (15 Gy in 1f) followed by nephrectomy	17.0	Not applicable	12 m OS 71% 24 m OS 47%	NR	Gd 3: 6% (anaemia)
(**b**)													
Cryotherapy													
Bang et al., 2012 [61]	OM	Single-arm	27 (72 lesions)	NR	NR	NR	TKI, IFN, bevacizumab, or other	Cryotherapy to metastases	16.0	LC 97%	Median OS 32.3 m	NR	Grade 3: 1.7%
Campbell et al., 2021 [46]	PM	Randomised feasibility	30	IMDC: favourable 14%, intermediate 68%, poor 17%	41% (all)	62%	Tremulimumab	Tremelimumab ± cryotherapy to metastases	29.0	NR	Median OS 22.7 m (cryo arm)	Median PFS 3 m (cryo arm)	Grade ≥ 3: 55% (cryo arm); cryoablation did not statistically enhance tremulimumab toxicity
Radiofrequency ablation													
Pellerin et al., 2013 [62]	PM	Single-arm	52 (58 lesions)	NR	NR	NR	NR	Embolisation, RFA, and cementoplasty to pelvic bone metastases	NR	LC NR; improvement in VAS score (*p* < 0.0001), QOL and narcotic use at 6 months	NR	NR	5 leakages to psoas (no clinical consequence), 1 temporary buttock claudication
Tsimafeyeu et al., 2013 [63]	CR	2× single-arm studies	38	MSKCC good 97%, intermediate 3%, high 0%	NR	97%	Sunitinib	RFA to primary kidney lesion	NR	ORR 29%	Median OS 27.2 m	Median PFS 13.4 m	Transient lumbar plexus pain (*n* = 11), perirenal haematoma (*n* = 1), inflammatory track mass (*n* = 2)
(**c**)													
Basket studies													
Spaas et al., 2021 (CHEERS abstract) [64]	PM	Phase 2 randomised	99 (SBRT 6 RCC, control 8 RCC)	NR	NR	NR	IO	IO vs. IO + SBRT to 1–3 lesions (24 Gy in 3f)	8.0 m control, 11.2 m SBRT	ORR 27% (no significant difference)	NR	Median PFS 4.4 m (no significant difference)	17.8% grade 3–4 toxicity (17.6% control)
Chang et al., 2007 [65]	NR	Phase 1/2 single-arm	63, 25 RCC (74 lesions)	NR	NR	NR	NR	SBRT to spine 30 Gy in 5f, 27 Gy in 3f (re-irradiation permitted)	21.3	LCR 77%	Median OS 24.3 m 12 m OS 69.8%	12 m PFS 84%	4.8% grade 3 toxicity
Ghia et al., 2016 (Garg 2012, Garg 2011) [66,67,68]	OM	2× single-arm (post hoc analysis)	43 RCC patients (47 lesions)	NR	NR	62.80%	NR	SBRT to spine (31.9% had prior spinal surgery). Single 24 Gy or multifraction (27 Gy in 3f or 30 Gy in 5f); re-irradiation permitted	23.0	LC 80.6 m 12 m LC 82% 24 m LC 68%	Median OS 22.8 m 12 m OS 74% 24 m OS 49%	NR	2.3% grade ≥ 3 toxicity (radiculopathy) 46% fracture (6/13 assessable sites) SF and 9.1% MF (1/11 assessable sites)

m—month, NR—not recorded, OM—oligometastatic, OP—oligoprogressive, PM—polymetastatic, CR—cytoreductive, IMDC—International Metastatic RCC Database Consortium, MSKCC—Memorial Sloan–Kettering Cancer Centre, CN—cytoreductive nephrectomy, RN—radical nephrectomy, TKI—tyrosine kinase inhibitor, IO—immunotherapy, ST—systemic therapy, IFN—interferon, BED—biologically effective dose, LC—local control, DCR—disease control rate, TTNT—time to next systemic treatment, ORR—overall response rate, OS—overall survival, PFS—progression-free survival, Gd—grade, DLT—dose-limiting toxicity, RT—radiotherapy, GFR—glomerular filtration rate, VAS—visual analogue score, SRS—stereotactic radiosurgery, SBRT—stereotactic body radiotherapy, Cryo—cryotherapy, RFA—radiofrequency ablation.

**Table 2:** Ongoing trials of metastasis-directed ablative therapy in both metastatic RCC and multiple histologies (basket).

Trial Identifier	Trial Title	Setting	Study Design		Status	Target Numbers	Estimated Completion	Primary Endpoint
RCC radiotherapy studies								
NCT05578664	PE-PE: Efficacy of perioperative pembrolizumab treatment in patients with resectable metastases from kidney cancer	OM	Randomised	Phase 2	Not yet recruiting	81	Oct-25	Relapse-free survival
NCT03575611	Stereotactic body radiation therapy in treating patients with oligometastatic renal cell carcinoma	OM	Single-arm	Feasibility	Recruiting	30	Sep-22	Feasibility and progression-free survival
NCT02956798	SAbR for oligometastatic renal cell carcinoma	OM	Single-arm	Phase 2	Active, not recruiting	23	Dec-23	Time to start of systemic therapy
NCT02542202	Stereotactic body radiation therapy in treating patients with metastatic or recurrent kidney cancer	OM or OR	Single-arm	Pilot	Recruiting	25	Jul-23	Grade 4 toxicity
NCT04299646	GETUG-StORM-01—Study assessing stereotactic radiotherapy in therapeutic strategy of oligoprogressive renal cell carcinoma metastases	OP	Randomised	Phase 2	Recruiting	114	Sep-23	Progression-free survival
NCT04974671	Trial of stereotactic body radiation therapy (SBRT) for oligoprogression on immune checkpoint inhibitors (ICI) in metastatic renal cell carcinoma	OP	Single-arm	Phase 2	Recruiting	30	Oct-27	Progression-free survival
NCT03696277	SAbR for oligoprogressive renal cell cancer	OP	Single-arm	Phase 2	Active, not recruiting	20	Oct-24	Time to change of systemic therapy
JPRN- UMIN000030972	NIVOSTRCC—Multi-institutional randomised phase 2 trial of a treatment of nivolumab combined with stereotactic body radiotherapy for patients with unresectable or metastatic renal cell carcinoma	PM	Randomised	Phase 2	Recruiting	100	Unknown	Response rate of nonirradiated lesion
NCT05567588	Pembrolizumab plus radiotherapy for advanced renal cancer	PM	Single-arm	Phase 2	Not yet recruiting	66	Oct-25	Objective response rate
NCT05327686	SAMURAI—Testing the addition of stereotactic radiation therapy with immune therapy for the treatment of patients with unresectable or metastatic renal cell cancer	CR	Randomised	Phase 2	Recruiting	240	Jun-32	Nephrectomy and progression-free survival
NCT04090710	CYTOSHRINK—SBRT with combination ipilimumab/nivolumab for metastatic kidney cancer	CR	Randomised	Phase 2	Recruiting	78	Dec-23	Progression-free survival
Basket radiotherapy studies								
NCT03862911	SABR-COMET-3—Phase 3 randomised controlled trial and economic evaluation of stereotactic ablative radiotherapy for comprehensive treatment of oligometastatic (1–3 metastases) cancer	OM (1–3)	Randomised	Phase 3 RCT	Recruiting	330	Dec-28	Overall survival
NCT03721341	SABR-COMET-10—A randomised phase 3 trial of stereotactic ablative radiotherapy for the comprehensive treatment of 4–10 oligometastatic tumours	OM (4–10)	Randomised	Phase 3 RCT	Recruiting	204	Jan-29	Overall survival
NCT04498767	OligoRARE—Stereotactic body radiotherapy in patients with rare oligometastatic cancers	OM	Randomised	Phase 3 RCT	Recruiting	200	Feb-30	Overall survival
NCT05259319	IMMUNOs-SBRT—Study evaluating the safety and the efficacy of combination of atezolizumab, tiragolumab and stereotactic body radiation therapy in patients with oligometastatic multiorgan	OM	Single-arm	Phase 1	Not yet recruiting	92	Feb-30	Grade 3 and grade 4 toxicity
NCT03599765	EXTEND: A randomised phase 2 basket trial assessing the efficacy of upfront local consolidative therapy (LCT) for oligometastatic disease	OM	Randomised	Phase 2	Recruiting	367	Dec-25	Incidence of adverse events
NCT04177056	SOLAR-P—Stereotactic body radiotherapy for osseous low alpha-beta resistant metastases for pain relief	PM	Single-arm	Cohort	Recruiting	40	Jan-23	Overall pain response
ChiCTR-IPR-17010456	Apatinib and radiotherapy compared with zoledronic acid and radiotherapy treatment in patients with bone metastases of malignant tumour	PM	Randomised	Phase 2	Recruiting	60	Unknown	Incidence of bone related events
Basket Thermal ablation studies								
NCT04375891	Radiation therapy alone versus radiation therapy plus radiofrequency ablation (RFA)/vertebral augmentation	PM	Randomised	Phase 2	Recruiting	80	May-26	Change in pain control
NCT04693377	CROME—Cryoablation combined with stereotactic body radiation therapy for the treatment of painful bone metastases	PM	Randomised	Phase 2	Recruiting	40	Apr-23	Pain response

**Table 3:** ROBINS-I tool for assessment of bias in nonrandomised studies. Abstracts were not able to be assessed [48,50,51,52,53,54,55,56,57,58,59,60,61,62,63,65,66,67,68].

Trial Author	Bias Due to Confounding	Bias of Selection	Bias in Classification of Interventions	Bias Due to Deviations from Intended Interventions	Bias Due to Missing Data	Bias in Measurement of Outcomes	Bias in Selection of Reported Result	Overall Bias
Bang et al., 2012 [61]	Moderate	Moderate	Low	Low	Low	Low	Low	Moderate
Pellerin et al., 2013 [62]	Moderate	Moderate	Low	Low	Low	Low	Low	Moderate
Tsimafeyeu et al., 2013 [63]	Low	Moderate	Low	Low	Low	Low	Low	Moderate
Tang et al., 2021 [48]	Moderate	Moderate	Low	Low	Low	Low	Low	Moderate
Siva et al., 2022 [49]	Moderate	Moderate	Low	Low	Low	Low	Low	Moderate
Hannan et al., 2021 [50]	Moderate	Moderate	Low	Low	Low	Low	Low	Moderate
Cheung et al., 2021 [51]	Moderate	Moderate	Low	Moderate	Low	Low	Low	Moderate
de Wolf et al., 2017 [52]	Low	Moderate	Low	Low	Low	Low	Low	Moderate
Masini et al., 2022 [53]	Moderate	Moderate	Low	Moderate	Low	Low	Low	Moderate
Dengina et al., 2019 [54]	Low	Moderate	Low	Low	Moderate	Low	Moderate	Moderate
Nguyen et al., 2010 [55]	Moderate	Moderate	Low	Low	Low	Low	Low	Moderate
Sohn et al., 2013 [56]	Moderate	Moderate	Low	Low	Low	Moderate	Moderate	Moderate
Svedman et al., 2009 [58]	Moderate	Moderate	Low	Low	Low	Moderate	Severe	Severe
Gerszten et al., 2005 [57]	Moderate	Moderate	Low	Low	Moderate	Moderate	Moderate	Moderate
Correa et al., 2018 [59]	Moderate	Moderate	Low	Low	Low	Low	Low	Moderate
Singh et al., 2022 [60]	Moderate	Moderate	Low	Low	Low	Low	Low	Moderate
Chang et al., 2007 [65]	Moderate	Moderate	Low	Low	Low	Low	Low	Moderate
Ghia et al., 2016 (Garg 2011, Garg 2012) [66,67,68]	Moderate	Moderate	Low	Low	Low	Low	Low	Moderate

## 4. Discussion

### 4.1. Main Results in the Context of Existing Literature

#### 4.1.1. Toxicity

There are existing data to support the low toxicity profiles of local ablative therapies inclusive of SBRT [37,38], RFA [40,69,70], and cryotherapy [26,71]. This review further supports this opinion. However, the majority of the studies utilised SBRT with grade ≥ 3 toxicity reported between 1.7% and 10% for local ablative therapy. This is comparable with retrospective data in mRCC ranging between 0% and 4.7% [40,72,73,74,75,76]. Furthermore, results are in keeping with a basket meta-analysis of prospective studies evaluating SBRT in OM, which reported grade ≥ 3 toxicity < 13% [37].

In this review, severe toxicities included but were not limited to anaemia, fatigue, pain, colitis, and hyperglycaemia. One study reported a death in a case of a large pleural lesion with SBRT, where the authors could not determine its relationship to treatment (see Table 1 [58]). No other local ablation-related deaths were reported. Inevitably, systemic combination studies reported higher grade ≥ 3 toxicity that ranged from 0% to 55%. Treatment toxicities are generally within the type and range expected from systemic therapy RCTs with grade ≥ 3 adverse effects ranging from 58.0% to 83.1% [2,3,4,5,6,7]. In a study of SBRT with systemic therapy in OP RCC, one patient had grade 3 colitis with small bowel perforation close to the radiation field 5 months post treatment alongside mTOR-I + TKI therapy. The authors reported that caution is required for SBRT in combination with systemic agents close to hollow organs such as the bowel [77]. However, no study reported any significant increases in severe toxicity that were definitively attributable to the addition of local ablative therapy.

The literature indicates that immediate post-procedural complications are more evident with RFA and cryoablation as these minimally invasive procedures require insertion of a needle or probe. Lung RFA carries a considerable risk of pneumothorax reported as 9–65% according to various case series [69,70,78]. Pneumothorax is a recognised complication of any needle procedure of the lung, with an overall pooled incidence for pneumothorax of 25.9% in a recent meta-analysis [79]. Post-procedural pain appears notable with cryoablation [26]. These procedures can be performed under local anaesthetic, but often require general anaesthetic, thus carrying the risk profile associated with any anaesthetic.

Vertebral compression fractures (VCFs) were reported at 15.3% and 0% in a multicentre, matched-pair study comparing spinal SBRT compared to fractionated radiotherapy in mRCC [56]. The SBRT dose per fraction was 10 Gy, which is lower than the previously reported threshold for VCF risk (around 20 Gy per fraction) [80,81]. In a post hoc analysis of 43 RCC patients from two phase 1–2 basket trials of SBRT for spinal metastases, post-treatment VCF was seen in 46% with single-fraction SBRT compared to 9.1% with multi-fraction SBRT [66]. Three out of these seven patients (42.9%) were asymptomatic and did not require intervention. This cohort included patients who had previous radiotherapy at the treated site i.e., re-irradiation, which is expected to carry a higher risk. However, VCF rates of 10–39% have been reported of patients receiving high-dose radiotherapy or SBRT with dose escalation correlating with increased risk [82,83,84,85]. The spinal instability neoplastic score (SINS) is likely to be a useful tool to assess fracture risk with radiotherapy [86]. Pre-emptive multi-modality or ‘hybrid’ treatment with surgical stabilisation, including vertebroplasty, in combination with SBRT of spinal metastases may offer the potential for better LC whilst limiting adverse effects and warrants further investigation.

#### 4.1.2. Local Control

Within this review, median LC at 12 months was 84.4%, (range 82.5–93) with a median LC rate of 87% (79–100%). This is comparable to a considerable number of retrospective mRCC SBRT studies, where the 12-month LC ranges from 70% to 98.2% [74,87,88,89,90,91]. Similar LC rates are also seen within a few retrospective studies of RFA in mRCC [92] and cryoablation studies [93].

#### 4.1.3. Overall Survival and Progression-Free Survival

In this review, of the studies that reported median OS, the combined median OS was 22.7 months (range 6.7–32.3 months) with a median follow-up of 22 months (range 13.1–29 months). The lower end of this OS range (6.7 months) was in the cytoreductive setting and was likely seen as this was a small phase 1 cohort of advanced inoperable tumours [59]. Median OS was not reached in one further study after a median follow-up of 10.4 months. However, these cohorts are small and are generally not powered sufficiently for survival outcomes. Regardless, these results are in line with retrospective data which indicate comparable or higher median OS ranging from 23.2 to 63.2 months [73,75,94,95,96].

This review reports a combined median PFS of 9.3 months (range 3.0–22.7 months) with a median follow-up of 14.7 months (range 10.4–29 months). Median PFS from retrospective studies suggests higher PFS benefits, with results ranging from 4.5 to 37.9 months [72,73,75,76,90,95]. However, these are unselected retrospective series with significant heterogeneity and, as such, were excluded from inclusion in this review.

### 4.2. Clinical Applicability

#### 4.2.1. Oligometastatic Setting

In this review, one cryotherapy and two SBRT studies for OM RCC demonstrated promising results (see Table 1a,b). In the two SBRT studies, all the patients had nephrectomy [48,49,61]. This is in keeping with SABR ORCA, a meta-analysis of SBRT in intracranial and extracranial OM RCC, which included 28 studies (27 retrospective and one prospective), comprising 1602 patients and 3892 lesions (1159 extracranial lesions) [97]. The 1-year LC was 89.1% (95% CI 83.6–93.7%, I^2^ = 71%), and 1-year survival rates were 86.8% (95% CI: 62–99.8%, I^2^ = 95%) [97]. The incidence of grade 3–4 toxicity was 0.7% (95% CI: 0–2.1%, I^2^ = 0%) for extracranial disease [97]. Furthermore, a meta-analysis of nine studies reviewing SBRT for spinal metastases from RCC reported 1-year LC rates of 71.2–85.7% with a significant improvement in comparison with conventional radiotherapy [85].

##### A ‘Radical’ Approach in Combination with Systemic Therapy

Long improvements in OS with first-line systemic therapy in clear cell mRCC have been demonstrated [2,3,4,5,6,7]. The RAPPORT study looked at ablating all mRCC lesions with SBRT alongside IO; all patients had nephrectomy [49] with promising disease control rate (DCR) of 83% after a median follow-up of 28 months, 24-month OS of 74%, and acceptable toxicity. Patients with limited low-volume metastatic disease may have a different natural history, whereby, if these lesions are ablated, then they may achieve longer remission without the need for long-term ongoing systemic therapy. In some patients, this may potentially lead to full disease eradication.

In a randomised pilot of tremelimumab with or without the addition of cryotherapy [46], 30 patients were enrolled; 14 received tremulimumab alone and 15 received additional cryotherapy (cryo-tremelimumab). One patient withdrew consent prior to treatment. A major limitation of this report was that anatomical sites treated were not listed. No patients had prior therapies and all patients had <5 sites of disease (median of two sites). The primary endpoint was safety, and 67% grade ≥ 3 toxicity was seen the combined arm with 43% in the control arm. This led to treatment discontinuation of 33.3% in the tremelimumab alone arm and 35.7% with cryo-tremelimumab. Only one objective response was seen in the combined treatment arm, and efficacy outcomes, although not formally powered, appeared to be inferior; the hazard ratio (HR) for cryo-tremelimumab versus tremelimumab was 1.68 (95% CI: 0.58, 4.87). In a post hoc analysis, patients with clear cell histopathology had more favourable changes in the immune microenvironment and a trend towards better clinical outcomes; 64% had clear cell in the control arm versus 60% in the cryo-tremulimumab arm.

##### Deferred ‘Consolidative’ Ablation or a Delay to Initiation of Systemic Therapy

There is also an argument to investigate deferred consolidative ‘radical’ local ablation to residual visible OM in patients who warrant upfront systemic therapy. This may be of considerable interest in the synchronous setting, i.e., in a cohort of patients that demonstrated good response to systemic therapy after a 3–6 month period.

A recent single-arm phase 2 feasibility study of 30 patients treated patients with OM clear cell RCC with SBRT to all lesions whilst maintaining patients off systemic therapy [48]. They reported that sequential radiotherapy may facilitate the deferral of initiation of systemic therapy and may allow for sustained systemic therapy breaks for select patients with OM RCC [98]. This is in keeping with a retrospective cohort of 47 patients which reported SBRT resulted in a freedom-from-systemic therapy interval of 15.2 months (95% CI, 8.8–40.1), and the duration of first-line treatment was subsequently unaffected by SBRT [99]. Toxicity from systemic therapy can be significant; as such, a radical local treatment approach without systemic therapy in carefully selected patients, whilst delaying time to initiating systemic treatment, could be of considerable value.

##### Results in Context of Metastasectomy and Active Surveillance Approaches

There are no randomised data identified to review local ablative therapies versus metastasectomy. Systematic reviews of retrospective data demonstrate excellent efficacy outcomes of metastasectomy; however, there is minimal prospective evidence. Metastasectomy, where feasible, is likely to be beneficial in fit patients particularly as part of a ‘radical’ approach in OM; complete resection appears to be a particularly important prognostic indicator along with a single site of disease [32]. Poor prognostic indicators may include higher pathological grade, >T3 stage primary, <12 months disease-free interval, and multi-organ involvement [100]. Definitive studies are warranted to evaluate efficacy and to guide patient-selection. Furthermore, the impact of recovery time from significant surgery and general anaesthetic is clinically important and should continue to be taken into account in multidisciplinary decision-making processes. This is particularly important in patients with a higher burden of disease or when in a second-line or more treatment setting, where a minimally invasive or non-invasive approach may be more appropriate.

AS appears to be a promising option for a select favourable group of patients described previously and warrants further prospective investigation [18,19]. However, prospective evidence of AS, to date, permits the use of metastasis-directed local therapies, which suggests that the benefit of this approach is likely to be in combination with effective local surgical and/or ablative techniques.

#### 4.2.2. Oligoprogressive Setting

##### A Delay to Change in Systemic Therapy or a Window for a Treatment-Break

In the OP setting, two SBRT studies were identified (no RFA and no cryotherapy studies); both studies included synchronous and metachronous metastatic patients. In a single-arm phase 2 clinical trial of 20 patients on first–fourth-line systemic therapy with ≤3 sites of OP, patients received upfront and sequential SBRT to a total of 37 sites. LC was reported at 100% [77]. At a median follow-up of 10.4 months, SBRT extended the duration of the ongoing systemic therapy by >6 months in 14 patients (70%, 95% CI: 49.9–90.1) [77]. In a multicentre single-arm study, 37 patients with 57 OP sites were treated with SBRT following at least 3 months of stable disease on TKI therapy [51]. The 1-year LC was 93% (95% CI 71–98%). The median PFS after SBRT was 9.3 months (95% CI 7.5–15.7). The median time to change in systemic therapy was 12.6 months (95% CI 9.6–17.4). There were no grade 3–5 radiotherapy-related toxicities [51].

This is in keeping with various retrospective studies, which highlight a potential benefit of this treatment strategy [71,101,102]. Metastasis-directed ablation may enable the ongoing systemic therapy to be used for longer and avoid the need to change systemic therapies where second-line or third-line therapies may be less effective and have more adverse effects. It may also offer the potential for a treatment break. After second-line systemic treatment, there is less evidence to guide management decisions [10,11]. Hence, more modest benefits in PFS are arguably much more clinically significant to patients in this setting than earlier in their treatment pathway. Local ablative therapies may offer the option for symptom control or to reduce the chance of development of debilitating sequelae of disease including spinal cord compression. In this way, it may reduce the requirement for palliative supportive care packages for disease progression.

#### 4.2.3. Polymetastatic Setting

In the PM setting, one cryotherapy, one RFA, and seven SBRT studies were identified. PM disease encompasses progression at many sites. There has been much interest in the ‘abscopal’ effect, i.e., an immunological phenomenon leading to response in distant metastatic disease after irradiation of a dominant lesion, particularly in the context of aiming to improve response rates with IO. The RADVAX study treated 1–2 sites of disease with SBRT alongside nivolumab and ipilumumab in a cohort of 25 patients. The overall response rate was promising at 56% with a median follow-up of 24 months [47]. This was in contrast to results from the phase 2, nonrandomised multicentre NIVES study [53]. A total of 69 patients with metastatic disease, who progressed after TKIs, received SBRT to one site of disease alongside nivolumab. Although no safety concerns arose, this study did not demonstrate a synergistic benefit [53]. The single-site approach has not yet been proven, and a move towards treating all lesions to see a maximal synergistic effect is gaining interest [103]. Further randomised studies are warranted.

#### 4.2.4. Cytoreductive Setting

In the CR setting, three studies (one RFA, one SBRT, and one SBRT + surgery) were identified. The use of cytoreductive nephrectomy (CN) was not validated in the phase 3 CARMENA [104] and SURTIME [105] studies; however, there may be a role in certain subgroups. The CARMENA trial included about 40% with poor-risk disease and 17% with deferred nephrectomy. The SURTIME trial demonstrated that deferred CN appears to be superior to immediate CN in terms of OS (median OS 32.4 months vs. 15.0 months, respectively). More patients received sunitinib in the deferred arm. These studies only included clear cell pathology and were not conducted in the era of IO.

Retrospective data have indicated that predictive factors for benefit for CN may include having <1 or <2 risk factors inclusive of IMDC risk factors (anaemia, high corrected calcium, neutrophilia, thrombocytosis, <80 Karnofsky performance status (KPS), and <1 year from diagnosis to treatment), locally advanced primary, nodal involvement, >3 metastases, and liver, lung, bone, and brain metastases [100].

Two RCTs, the PROBE trial (NCT04510597) and NORDIC-SUN (NCT03977571), are underway to investigate deferred CN. CR SBRT of the primary may be a good non-invasive option for patients who are unfit for surgical options. However, there are minimal prospective data available to evaluate this approach [59,60]. There are similarly little data in this setting investigating RFA and no prospective data for cryotherapy [63].

### 4.3. Prognostic and Predictive Factors

Patients within eligible studies were generally of good performance status (PS): Eastern Cooperative Oncology Group (ECOG) PS 0–1. Retrospective data suggest that performance status may also be a prognostic indicator for survival outcomes [73,74,95]. Metachronous metastatic disease has been suggested to have a more favourable prognosis than de novo or synchronous mRCC [99]. However, in a phase 2 study of SBRT for OP in a cohort of 20 patients alongside systemic therapy, 60% of which had synchronous metastatic disease, a statistically significant difference was observed in 6 month PFS for patients with five or fewer metastases at the time of SBRT compared with that for patients with six or more metastases (100% vs. 50%; *p* = 0.0037) [77]. This is in line with existing data where low-volume disease has been commonly noted as prognostic for survival outcomes [74,95]. Hence, definitive investigation of an ablative approach in synchronous mRCC alongside systemic therapy is also warranted.

Over two-thirds of patients were intermediate-risk in eligible studies, and the median proportion of clear cell patients was 93.5% (range 62–100) [see Table 1a,b]. In a multicentre retrospective study of SBRT, 70 patients with 1–5 OM (78.4% lung metastases) were treated with the aim of delaying a switch in systemic therapy [76]. Interestingly, clear cell pathology on multivariate analysis was a predictive indicator of poor OS compared to non-clear cell histology. Furthermore, in another retrospective study of SBRT, worse IMDC and sarcomatoid pathology did not appear to demonstrate a difference in efficacy outcomes [73]. This suggests that ablation may be able to overcome relative resistance clones of non-clear cell pathology, as well as poor-risk groups, and such patients may also benefit from SBRT.

A prospective study of 17 patients treated with SBRT with systemic therapy in mRCC reported that a fraction size of ≥10 Gy or equivalent dose in 2 Gy per fraction (EQD2) of ≥100 Gy was associated with a significant increase in complete response observed [54]. This in keeping with the existing literature, which suggests that dose escalation or a BED ≥100 Gy is required in RCC for effective cancer control [76,90,92,106]. Certain anatomical sites of metastases are likely to benefit less from ablative therapy, particularly if close to critical surrounding normal tissues which limit the dose of radiotherapy or feasibility of thermal ablation. The spinal site has been suggested to be associated with higher local failures in a number of retrospective studies [92,99,102]. In primary RCC, RFA in smaller tumour size may have improved outcomes [107].

Favourable local response has been suggested as a potential future surrogate for survival benefit [73]. In another retrospective study of SBRT with IO, 85.1% had one prior therapy only, patients with a median duration of first-line therapy ≥8.6 months had significantly longer OS (38.5 vs. 16.2 months; *p* = 0.041) [94].

### 4.4. Overall Completeness and Applicability of Evidence

Due to the use of three separate source resources, the authors expect that this systematic review identified all of relevant studies as per protocol eligibility criteria. The evidence overall involved small prospective non-randomised cohorts (12 of the 18 studies had 30 or fewer patients), and there was inconsistency in terms of treatment regimes, median follow-up, and endpoint reporting. Only four eligible thermal ablation studies were identified, and there were no eligible studies analysing the role of microwave ablation in this setting. Due to RCC not being a very common cancer, and due to these techniques requiring significant tertiary centre expertise, pooling such evidence is important, in order to guide future development of translationally-rich randomised interventional studies which aim to answer definitively the question of efficacy in various clinical settings.

### 4.5. Quality of the Evidence

The quality of individual studies was level 3 as the majority were non-randomised controlled cohorts as per the Oxford Centre for Evidence-Based Medicine criteria (OCEBM Levels of Evidence Working Group ‘The Oxford 2011 Levels of Evidence’).

### 4.6. Potential Biases in the Review Process

During the review process, as one author was involved in the initial screening process, this may have increased the chance of bias in title review. However, all cases that were considered borderline for inclusion and all full texts were discussed among authors.

### 4.7. Agreements and Disagreements with Other Studies or Reviews

This systematic review is in line with the current international consensus that SBRT appears to be a safe and effective treatment for LC in metastatic extracranial RCC. However, the true effect size and criteria for patient selection need to be evaluated through high-quality randomised studies. No systematic reviews of thermal ablation in mRCC were identified for comparison.

## 5. Conclusions and Future Directions

This systematic review reports that SBRT is safe, is feasible, and provides excellent local control in RCC. There were <5 eligible thermal ablative studies identified in this setting; as such, comparisons with SBRT were not feasible. Although there is a lack of prospective data for thermal ablative techniques for mRCC, they are widely used amongst other tumour types, which have demonstrated both their acceptable safety profile and their excellent local control outcomes.

This review highlighted that there are key questions that remain unanswered. Definitive studies are required to evaluate the additional efficacy of local ablative therapy compared to current standard management and to identify which patient groups are likely to benefit the most. Primary cancer histology may impact the clinical benefit of such ablative treatments; as such, RCTs with representative proportions of RCC participants are required to address the question in this disease setting. Study designs require development with ongoing patient and public involvement. Integration of patient-reported outcome measures will aim to ensure that our use of such techniques results in patient-relevant outcome benefits. Future work should also consider the benefit of multimodality treatments, as this is an under-investigated option that is anticipated to potentially translate to clinical benefit in the future.

## Supplementary Materials

The following supporting information can be downloaded at: https://www.mdpi.com/article/10.3390/cancers15133455/s1, File S1: The PRISMA checklist and list of acronyms is supplied.
