# Supplementary material for: Beyond the Knife in Renal Cell Carcinoma: A Systematic Review—To Ablate or Not to Ablate?
Source: Cancers (Basel). 2023 Jun 30;15(13):3455. doi: 10.3390/cancers15133455 (PMC10340567; doi:10.3390/cancers15133455)
Supplement: Supplementary file 1 [file cancers-15-03455-s001.zip › cancers-2457335-supplementary/List of Acronyms.pdf]

Acronyms

4DCT	4-dimensional computerised tomography
AS	Active Surveillance
BED	Biologically effective dose
CI	Confidence Interval
CN	Cytoreductive nephrectomy
CNS	Central nervous system
DCR	Disease control rate
DFS	Disease-free survival
DNA	Deoxyribonucleic Acid
ECOG	Eastern Cooperative Oncology Group
EQD2	Equivalent dose in 2Gy per fraction
FR	Favourable risk
HR	Hazard Ratio
IMDC	International Metastatic RCC Database Consortium
IL2	Interleukin-2
IO	Immunotherapy
IR	Intermediate-risk
KPS	Karnofsky Performance Status
LC	Local Control
m	Month(s)
mBC	Metastatic Breast Cancer
mNSCLC	Metastatic Non-Small Cell Lung Cancer
mRCC	Metastatic RCC
MSKCC	Memorial Sloan-Kettering Cancer Centre RCC Score
MTD	Maximum Tolerated Dose
mTOR-I	Mammalian Target Of Rapamycin-inhibitors
OM	Oligometastatic/Oligometastases
OP	Oligoprogressive/Oligoprogression
ORR	Overall Response Rate
OS	Overall Survival
PFS	Progression-free survival
PM	Polymetastatic/Polymetastases
PR	Poor-risk
PRISMA	Preferred Reporting Items for Systematic Reviews and Meta-Analyses
PROMs	Patient-Reported Outcome Measures
PS	Performance Status
RCC	Renal cell carcinoma
RCT	Randomised Controlled Trial
RFA	Radiofrequency ablation
SBRT	Stereotactic body radiotherapy
SINS	Spinal instability neoplastic score
SOC	Standard of care
TKI	Tyrosine Kinase Inhibitors
TTNT	Time to next systemic treatment
VAS	Visual analogue score
VCF	Vertebral Compression Fracture
VMAT	Volumetric-modulated arc therapy
